# Elevated plasma D-dimer levels in patients with anti-N-methyl-D-aspartate receptor encephalitis

**DOI:** 10.3389/fneur.2022.1022785

**Published:** 2022-11-15

**Authors:** Yingzhe Shao, Juan Du, Yajun Song, Yanfei Li, Lijun Jing, Zhe Gong, Ranran Duan, Yaobing Yao, Yanjie Jia, Shujie Jiao

**Affiliations:** Department of Neurology, The First Affiliated Hospital of Zhengzhou University, Zhengzhou, China

**Keywords:** anti-N-methyl-D-aspartate receptor encephalitis, D-dimer, inflammation, neutrophil, eosinophils, calcium

## Abstract

**Purpose:**

We aimed to explore the difference in coagulation function between healthy individuals and patients with anti-N-methyl-D-aspartate receptor (anti-NMDAR) encephalitis and its relationship with disease severity.

**Methods:**

We retrospectively compared coagulation function in 161 patients with first-attack anti-NMDAR encephalitis and 178 healthy individuals. The association between D-dimer levels and disease severity was analyzed using binary logistic regression. Receiver operating characteristic (ROC) curves were used to analyze the predictive value of D-dimer levels for the severity of anti-NMDAR encephalitis.

**Results:**

Compared to control individuals, patients with anti-NMDAR encephalitis had higher D-dimer levels (median 0.14 vs. 0.05 mg/L, *p* < 0.001), blood white blood cell (WBC) count (median 8.54 vs. 5.95 × 10^9^/L, *p* < 0.001), and neutrophil count (median 6.14 vs. 3.1 × 10^9^/L, *p* < 0.001). D-dimers (median 0.22 vs. 0.10 mg/L, *p* < 0.001), blood WBC count (median 9.70 vs. 7.70 × 10^9^/L, *p* < 0.001), neutrophil count (median 7.50 vs. 4.80 × 10^9^/L, *p* < 0.001), and C-reactive protein (median 2.61 vs. 1.50 mg/l, *p* = 0.017) were higher; however, eosinophils (median 0.02 vs. 0.06 × 10^9^/L, *p* < 0.001), and blood calcium (median 2.26 vs. 2.31 mmol/L, *p* = 0.003) were lower in patients with severe forms of anti-NMDAR encephalitis than in those with mild to moderate forms, and were associated with initial modified Rankin Scale scores. Multivariate analysis showed that D-dimer levels were significantly associated with severity [odds ratio =2.631, 95% confidence interval (CI) = 1.018–6.802, *p* = 0.046]. The ROC curve was used to analyze the predictive value of D-dimer levels for disease severity. The area under the curve was 0.716 (95% CI = 0.64–0.80, *p* < 0.001), and the best cut-off value was D-dimer = 0.147 mg/L (sensitivity 0.651; specificity, 0.705).

**Conclusion:**

Serum D-dimer and neutrophil levels were independent predictors of disease severity in patients with first-attack anti-NMDAR encephalitis.

## Introduction

Autoimmune encephalitis is a class of encephalitis caused by an immune response to central nervous system antigens mediated by autoimmune mechanisms. Among these, anti-N-methyl-D-aspartate receptor (anti-NMDAR) encephalitis is the most common type, which places a huge economic burden on patients (1). The main clinical symptoms are abnormal (psychiatric) behaviors, cognitive dysfunction, speech disorders, epilepsy, motor disorders, reduced awareness, and autonomic nerve dysfunction, etc. (2). Currently, few biomarkers can felicitously assess the severity of anti-NMDAR encephalitis.

Routine blood and coagulation functions are the most common laboratory indicators in clinics. However, white blood cells (WBC), neutrophils and C-reactive protein (CRP) are also indicators of systemic inflammation. D-dimer is a soluble fibrin degradation product, the expression of which elevated in arterial or venous thrombosis, disseminated vascular coagulation, advanced age, surgery, trauma, tumors, pregnancy, infection, chronic inflammation, liver disease, and kidney disease (3–5). Recent studies have found that D-dimer is associated with disease severity in autoimmune diseases such as lupus (6), rheumatoid arthritis (7), and granulomatosis with polyangiitis (8). In addition, several recent studies have highlighted the importance of activating the coagulation cascade in neuro-inflammation (9–11). Therefore, coagulation tests may be useful in identifying severe cases of anti-NMDAR encephalitis. No clinical research on anti-NMDAR encephalitis and coagulation function has been conducted to date. The aim of the present study was to investigate the differences in coagulation function between healthy people and patients with anti-NMDAR encephalitis and their relationship with disease severity.

## Methods

### Patients

This study was ratified by the Ethics Committee of Zhengzhou University (2019-KY-018). In this retrospective study, we registered 230 patients with first-attack anti-NMDAR encephalitis who were treated at the First Affiliated Hospital of Zhengzhou University between April 2014 and April 2021, all of whom met the standard of diagnosis for anti-NMDAR encephalitis (12) (Chinese expert consensus on the diagnosis and management of autoimmune encephalitis 2017).

**Figure 1:**
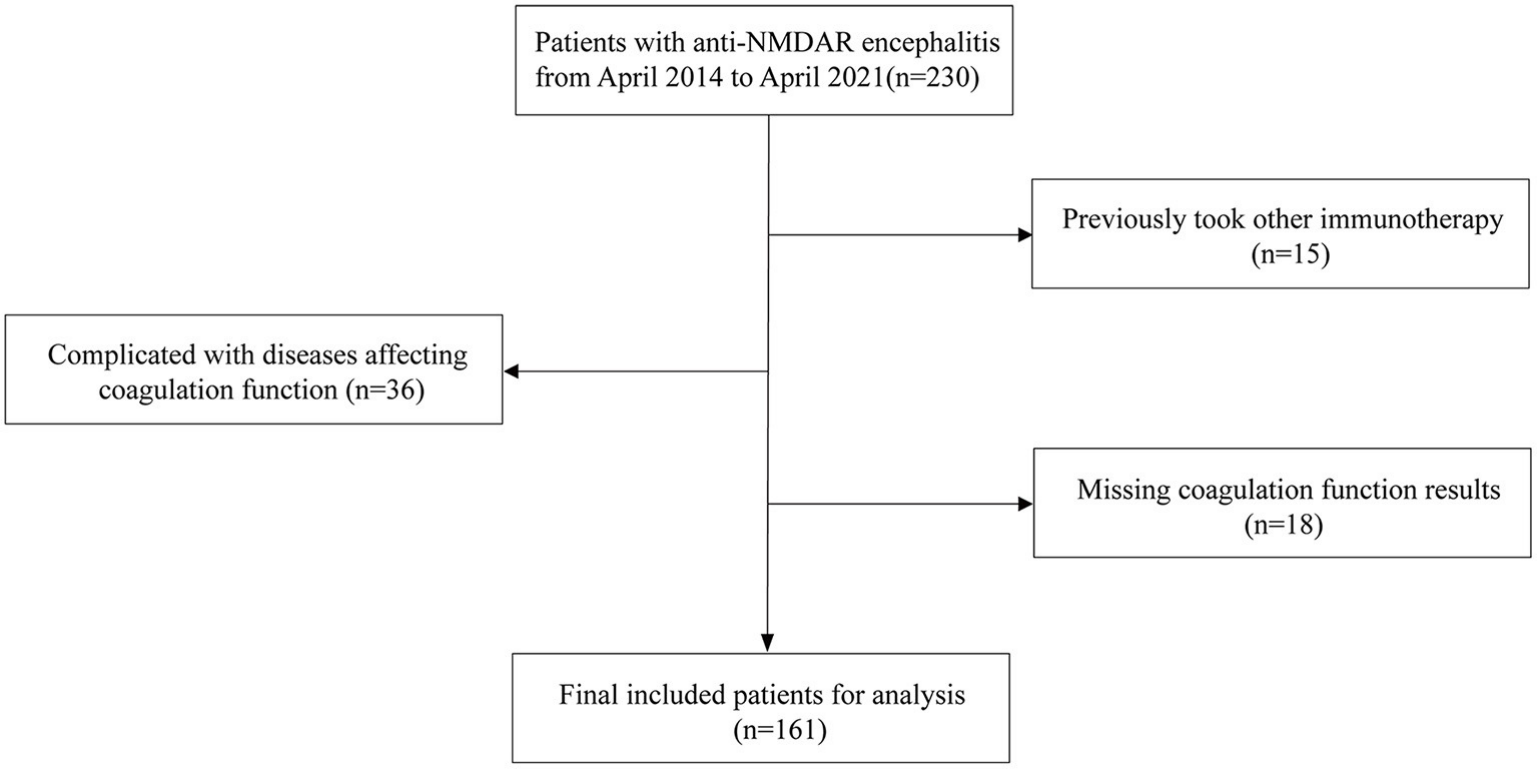
Patients selection process. Anti-NMDAR encephalitis, anti-N-methyl-D-aspartate receptor encephalitis.

The exclusion criteria were as follows: (i) complications with tumors, severe liver and kidney dysfunction, blood system diseases, arteriovenous thrombosis, severe infection, and other diseases affecting coagulation function; (ii) immunotherapy such as plasma exchange, corticosteroids, intravenous immunoglobulin, and immunity inhibitor within 6 months before admission; (iii) anticoagulant thrombolytic therapy within 3 months before admission; (iv) age > 80 years; and (v) missing coagulation function results or inconsistent test reporting units. Gender- and age-matched healthy individuals (*n* = 178) were comprised in the control group. In total, 161 patients with anti-NMDAR encephalitis and 178 control individuals were included in this study (Figure 1).

### Data collection

Data related to baseline clinical information, demographic characteristics, medical history, clinical symptoms, laboratory test results, and imaging characteristics were collected from the case reports.

Blood samples were collected on an empty stomach during the early hours of the 2nd day after admission. Coagulation function, CRP, and blood calcium were tested and routine blood tests were performed in the biochemical laboratory of the First Affiliated Hospital of Zhengzhou University. Blood WBC count (3.5–9.5 × 10^9^/L), red blood cell (RBC) count (3.8–5.1 × 10^12^/L), platelet count (125–350 × 10^9^/L), hemoglobin (115–150 g/L), neutrophil count (1.8–6.3 × 10^9^/L), lymphocyte count (1.1–3.2 × 10^9^/L), monocyte count (0.1–0.6 × 10^9^/L), eosinophil count (0.02–0.52 × 10^9^/L), basophil count (0–0.06 × 10^9^/L), prothrombin time (PT; 8.8–13.6 s), activated partial thrombin time (APTT; 26–40 s), fibrinogen (2–4 g/L), thrombin time (TT; 10–18 s), D-dimer (0–0.3 mg/L DDU), CRP (0–10 mg/L), and blood calcium (2.0–2.7 mmol/L) was performed. Blood counts were analyzed using an automated analyzer. PT, APTT, TT, and fibrinogen were coagulation markers, and D-dimer was used for immune turbidimetry. Scatter turbidimetry was used to measure the CRP levels. Calcium ion colorimetry was also performed.

Cerebrospinal fluid analysis and cerebral magnetic resonance imaging (MRI) were performed prior to treatment. Serum and cerebrospinal fluid samples from patients were used to determine their anti-NMDAR status at the Neurology Laboratory of the First Affiliated Hospital of Zhengzhou University using cell-based assays. MRI was performed using a 3.0 T Philips Healthcare (Best, Netherlands).

At least two professional neurologists carefully reviewed the patients' clinical records to calculate the extended modified Rankin Scale (m-RS) scores at admission and discharge. The initial and discharge m-RS scores were recorded, and the former was used to evaluate disease severity. The m-RS scores were converted into categorical variables. Patients with an initial m-RS score of >3 at admission were defined as the severe group, and those with an initial m-RS score ≤3 were defined as the mild-to-moderate group.

First-line immunotherapy includes plasma exchange, corticosteroids, and intravenous immunoglobulin; second-line immunotherapy includes rituximab and intravenous cyclophosphamide; and long-term immunotherapy includes mycophenolate mofetil and azathioprine.

### Statistical analysis

Data were analyzed using SPSS software (version 26.0; International Business Machines Corporation, Chicago, IL, USA). Graphs were generated by GraphPad Prism 8.3 (GraphPad Inc., Harvey Motulsky, USA).

Normality tests were performed using the Kolmogorov-Smirnov test. If the continuous variables fit a normal distribution, they were expressed as means and standard deviations (SD) and compared using *t*-tests. Non-normally distributed data were presented as medians (interquartile ranges) and were contrasted using the Mann-Whitney *U*-test. Categorical data were presented as frequency (percentage), and the chi-square test or Fisher's exact test was used for comparison between groups. Spearman's correlation coefficient was calculated to assess the correlations. Univariate and multivariate analyses were used to evaluate the factors potentially related to disease severity in first-attack anti-NMDAR encephalitis patients. The receiver operating characteristic (ROC) curve was used to calculate the predictive value of D-dimer levels for disease severity. All tests were two-sided with a significance level of 0.05.

## Results

### Clinical characteristics of the patients with anti-NMDAR encephalitis and healthy individuals

Seventy-three (45.34%) female patients aged 25 years on average (16–37) were included in the anti-NMDAR encephalitis group (*n* = 161). In the control group (*n* = 178), 85 female patients (47.75%) aged 27 years on average (17–39.25) were included. There were no obvious differences in age or gender between the two groups (*p* > 0.05), indicating comparability. The platelet count and incidence of hypertension and diabetes did not differ significantly between the two groups. Our findings indicated that PT (median 11.0 vs. 10.5 s, *p* < 0.001), fibrinogen (median 2.78 vs. 2.45 g/L, *p* < 0.001), D-dimer (median 0.14 vs. 0.05 mg/L, *p* < 0.001), blood WBC count (median 8.54 vs. 5.95 × 10^9^/L, *p* < 0.001), neutrophil count (median 6.14 vs. 3.10 × 10^9^/L, *p* < 0.001), and monocyte count (median 0.56 vs. 0.41 × 10^9^/L, *p* < 0.001) were higher in patients with anti-NMDAR encephalitis than in healthy individuals. In contrast, the APTT levels (median 32.1 vs. 28.8 s, *p* < 0.001), TT (median 14.95 vs. 14.60 s, *p* = 0.021), RBC count (average 4.53 vs. 4.34 × 10^12^/L, *p* < 0.001), hemoglobin (average 135.37 vs. 130.20 g/L, *p* < 0.001), lymphocyte count (median 2.02 vs. 1.56 × 10^9^/L, *p* < 0.001), eosinophil count (median 0.11 vs. 0.04 × 10^9^/L, *p* < 0.001), and basophil count (median 0.03 vs. 0.03 × 10^9^/L, *p* = 0.026) were significantly higher in healthy controls than in patients with anti-NMDAR encephalitis (Table 1).

**Table 1:** Clinical characteristics of the patients with anti-NMDAR encephalitis and healthy controls.

**Clinical characteristics**	**Anti-NMDAR encephalitis** ** (*n* = 161)**	**Control** ** (*n* = 178)**	***p***
Age, years, median (IQR)	25 (16–37)	27 (17–39.25)	0.623
Gender, female, *n* (%)	73 (45.34)	85 (47.75)	0.657
Hypertension, *n* (%)	14 (8.70)	11 (6.18)	0.376
Diabetes, *n* (%)	4 (2.48)	3 (1.68)	0.605
PT, median (IQR; s)	11.0 (10.2–11.6)	10.5 (10.1–11.1)	<0.001^*^
APTT, median (IQR; s)	28.8 (26.05–31.45)	32.1 (29.68–34.50)	<0.001^*^
Fibrinogen, median (IQR; g/L)	2.78 (2.32–3.35)	2.45 (2.17–2.89)	<0.001^*^
TT, median (IQR; s)	14.60 (13.45–16.00)	14.95 (14.18–16.03)	0.021^*^
D-dimer, median (IQR; mg/L)	0.14 (0.07–0.36)	0.05 (0.038–0.08)	<0.001^*^
WBC, median (IQR; 10^9^/L)	8.54 (6.60–11.60)	5.95 (4.96–6.90)	<0.001^*^
RBC, mean ± SD (10^12^/L)	4.34 ± 0.53	4.53 ± 0.48	0.001^*^
Hemoglobin, mean ± SD (g/L)	130.20 ± 16.55	135.37 ± 15.00	0.001^*^
Platelet, median (IQR; 10^9^/L)	240 (197–308)	233 (190.75–282.25)	0.15
Neutrophil, median (IQR; 10^9^/L)	6.14 (3.96–8.91)	3.10 (2.55–4.00)	<0.001^*^
Lymphocyte, median (IQR; 10^9^/L)	1.56 (1.13–2.46)	2.02 (1.65–2.46)	<0.001^*^
Monocytes, median (IQR; 10^9^/L)	0.56 (0.43–0.75)	0.41 (0.35–0.51)	<0.001^*^
Eosinophils, median (IQR; 10^9^/L)	0.04 (0.01–0.10)	0.11 (0.07–0.18)	<0.001^*^
Basophil, median (IQR; 10^9^/L)	0.03 (0.01–0.04)	0.03 (0.02–0.04)	0.026^*^

Normally distributed variables were presented as mean ± SD and compared using t-tests. Non-normally distributed variables were presented as median (IQR = 25–75th percentile) and were contrasted using the Mann-Whitney U-test. Categorical variables were described as number (percentage), and the chi-square test or Fisher's exact test was used for comparison between groups.

Anti-NMDAR encephalitis, anti-N-methyl-D-aspartate receptor encephalitis; PT, prothrombin time; APTT, activated partial thrombin time; TT, thrombin time; WBC, white blood cell; RBC, red blood cell.

* *p* < 0.05.

### Clinical characteristics of the patients with anti-NMDAR encephalitis with different severity

In the current study, the severe group had a higher proportion of female patients (53.01 vs. 37.18%, *p* = 0.044), intensive care unit admission rates (84.34 vs. 23.08, *p* < 0.001), and longer hospital stays (median 32 vs. 24, *p* = 0.025). Furthermore, patients in the severe group exhibited a significantly higher incidence of decreased consciousness (79.52 vs. 10.26 %, *p* < 0.001), abnormal (psychiatric) behaviors (89.16 vs. 60.26%, *p* < 0.001), movement disorders (54.22 vs. 21.79%, *p* < 0.001), and autonomic dysfunction (39.76 vs. 19.23%, *p* = 0.004) than those in the mild to moderate group. The PT (median 11.25 vs. 10.84 s, *p* = 0.027), D-dimer (median 0.22 vs. 0.10 mg/L, *p* < 0.001), blood WBC count (median 9.70 vs. 7.70 × 10^9^/L, *p* < 0.001), neutrophil count (median 7.50 vs. 4.80 × 10^9^/L, *p* < 0.001), and CRP (median 2.61 vs. 1.50 mg/l, *p* = 0.017) in the severe group was higher than that in the mild to moderate group (hemoglobin: median 128.00 vs. 134.00 g/L, *p* = 0.026; lymphocytes: median 1.43 vs. 1.70 × 10^9^/L, *p* = 0.002; eosinophils: median 0.02 vs. 0.06 × 10^9^/L, *p* < 0.001, and blood calcium: median 2.26 vs. 2.31 mmol/L, *p* = 0.003). The difference in APTT, fibrinogen, RBC, platelets, monocytes, and basophils between the two groups was insignificant. In addition, significant differences in m-RS between the two groups were present at discharge (median 2 vs. 1, *p* < 0.001), along with insignificant differences in MRI abnormality proportion, and treatment options, as shown in Table 2.

**Table 2:** Clinical characteristics of the patients with anti-NMDAR encephalitis severe group and mild to moderate group.

**Clinical characteristics**	**Total**	**Mild to moderate group**	**Severe group**	***p***
	**(*n* = 161)**	**(*n* = 78)**	**(*n* = 83)**	
Age at onset, years, median (IQR)	25 (16–37)	27 (17.75–42)	24 (15–33)	0.283
Gender, female, *n* (%)	73 (45.34)	29 (37.18)	44 (53.01)	0.044^*^
Hospital stays, days, median (IQR)	26 (18–41)	24 (17.75–35.25)	32 (18–48)	0.025^*^
ICU admission, *n* (%)	88 (54.66)	18 (23.08)	70 (84.34)	<0.001^*^
Hypertension, *n* (%)	14 (8.70)	9 (11.54)	5 (6.02)	0.215
Diabetes, *n* (%)	4 (2.48)	2 (2.56)	2 (2.41)	1
Decreased level of consciousness, *n* (%)	74 (45.96)	8 (10.26)	66 (79.52)	<0.001^*^
Abnormal (psychiatric) behavior, *n* (%)	121 (75.16)	47 (60.26)	74 (89.16)	<0.001^*^
Cognitive dysfunction, *n* (%)	73 (45.34)	34 (43.59)	39 (46.99)	0.665
Speech dysfunction, *n* (%)	72 (44.72)	29 (37.18)	43 (51.81)	0.062
Movement disorder, *n* (%)	62 (38.51)	17 (21.79)	45 (54.22)	<0.001^*^
Seizures, *n* (%)	105 (65.22)	45 (57.69)	60 (72.29)	0.052
Autonomic dysfunction, *n* (%)	48 (29.81)	15 (19.23)	33 (39.76)	0.004^*^
PT, mean ± SD (s)	11.08 ± 1.16	10.84 ± 0.89	11.25 ± 1.31	0.027^*^
APTT, median (IQR; s)	28.75 (26.13–31.43)	29.4 (27.275–32)	28.1 (25.675–31.325)	0.068
Fibrinogen, median (IQR; g/L)	2.85 (2.40–3.41)	2.88 (2.25–3.51)	2.85 (2.45–3.25)	0.266
TT, median (IQR; s)	14.45 (13.30–15.93)	14.60 (13.23–16.00)	14.40 (13.45–15.75)	0.351
D-dimer, median (IQR; mg/L)	0.15 (0.08–0.40)	0.10 (0.05–0.19)	0.22 (0.12–0.54)	<0.001^*^
WBC, median (IQR; 10^9^/L)	8.53 (6.60–11.58)	7.70 (6.05–9.50)	9.70 (7.32–12.90)	<0.001^*^
RBC, mean ± SD (10^12^/L)	4.35 ± 0.53	4.39 ± 0.54	4.31 ± 0.52	0.307
Hemoglobin, median (IQR; g/L)	130.30 (119.03–139.75)	134.00 (125.15–144.60)	128.00 (119.00–135.00)	0.026^*^
Platelet, median (IQR; 10^9^/L)	239.5 (197–307.75)	245 (199.5–307.5)	238 (192–310)	0.489
Neutrophil, median (IQR; 10^9^/L)	6.13 (3.98–8.89)	4.80 (3.59–7.45)	7.50 (4.70–10.80)	<0.001^*^
Lymphocyte, median (IQR; 10^9^/L)	1.56 (1.13–2.20)	1.70 (1.40–2.23)	1.43 (0.78–2.05)	0.002^*^
Monocytes, median (IQR; 10^9^/L)	0.56 (0.43–0.75)	0.52 (0.42–0.70)	0.59 (0.45–0.77)	0.249
Eosinophils, median (IQR; 10^9^/L)	0.04 (0.01–0.10)	0.06 (0.03–0.13)	0.02 (0.01–0.07)	<0.001^*^
Basophil, median (IQR; 10^9^/L)	0.03 (0.01–0.04)	0.03 (0.02–0.05)	0.03 (0.01–0.04)	0.055
Calcium, median (IQR; mmol/L)	2.29 (2.21–2.38)	2.31 (2.26–2.42)	2.26 (2.17–2.35)	0.003^*^
CRP, median (IQR; mg/L)	1.73 (0.79–7.03)	1.50 (0.50–5.00)	2.61 (1.00–8.32)	0.017^*^
Initial m-RS score, median (IQR)	4 (3–5)	3 (2–3)	5 (4–5)	<0.001^*^
Discharge m-RS score, median (IQR)	2 (1–3)	1 (1–2)	2 (1–4)	<0.001^*^
MRI abnormality, *n* (%)	62 (44.60)	31 (44.29)	31 (44.93)	0.939
First-line immunotherapy, *n* (%)	156 (96.89)	75 (96.15)	81 (97.59)	0.957
Second-line immunotherapy, *n* (%)	9 (5.59)	3 (3.85)	6 (7.23)	0.497
Long-time immunotherapy, *n* (%)	15 (9.32)	7 (8.97)	8 (9.64)	0.885

Normally distributed variables were presented as mean ± SD and compared using t-tests. Non-normally distributed variables were presented as medians (IQR = 25–75th percentile) and were contrasted using the Mann-Whitney U-test. Categorical variables were described as number (percentage), and the chi-square test or Fisher's exact test was used for comparison between groups. Severe group, patients with an initial m-RS score of >3 at admission; mild-to-moderate group, patients with an initial m-RS score ≤ 3.

Anti-NMDAR encephalitis, anti-N-methyl-D-aspartate receptor encephalitis; ICU, intensive care unit; PT, prothrombin time; APTT, activated partial thrombin time; TT, thrombin time; WBC, white blood cell; RBC, red blood cell; CRP, C-reaction protein; m-RS, modified Rankin Scale scores.

* *p* < 0.05.

### Correlations between D-dimer and disease severity in patients with anti-NMDAR encephalitis

Spearman correlation analysis showed that PT (*r* = 0.160, *p* = 0.042) and D-dimer levels (*r* = 0.367, *p* < 0.001), WBC count (*r* = 0.311, *p* < 0.001), hemoglobin (*r* = −0.188, *p* = 0.017), neutrophils (*r* = 0.340, *p* < 0.001), lymphocytes (*r* = −0.266, *p* < 0.001), eosinophils (*r* = −0.310, *p* < 0.001), basophils (*r* = −0.163, *p* = 0.039), calcium (*r* = −0.268, *p* = 0.001), CRP (*r* = 0.206, *p* = 0.016), and the female ratio (*r* = −0.164, *p* = 0.038) were significantly correlated with the initial m-RS score of patients with anti-NMDAR encephalitis. There was no obvious correlation between age at onset, APTT, fibrinogen, TT, RBC count, platelet count, monocyte count, and disease severity (*p* > 0.05; Table 3).

**Table 3:** Spearman correlation analysis between D-dimer and i-mRS in patients with anti-NMDAR encephalitis.

	**i-mRS**	
	***r***	***p***
Age at onset	−0.132	0.096
Gender	−0.164	0.038^*^
PT	0.160	0.042^*^
APTT	−0.115	0.148
Fibrinogen	0.004	0.965
TT	−0.037	0.644
D-dimer	0.367	<0.001^*^
WBC	0.311	<0.001^*^
RBC	−0.109	0.168
Hemoglobin	−0.188	0.017^*^
Platelet	−0.070	0.380
Neutrophil	0.340	<0.001^*^
Lymphocyte	−0.266	0.001^*^
Monocytes	0.152	0.055
Eosinophils	−0.310	<0.001^*^
Basophil	−0.163	0.039^*^
Calcium	−0.268	0.001^*^
CRP	0.206	0.016^*^

Anti-NMDAR encephalitis, anti-N-methyl-D-aspartate receptor encephalitis; PT, prothrombin time; APTT, activated partial thrombin time; TT, thrombin time; WBC, white blood cell; RBC, red blood cell; CRP, C-reaction protein; i-mRS, initial modified Rankin Scale scores.

* *p* < 0.05.

Univariate logistic regression analysis showed that the gender (OR = 1.906, 95% CI = 1.015–3.579, *p* = 0.045), PT (OR = 1.332, 95% CI = 1.027–1.726, *p* = 0.031), D-dimer (OR = 2.557, 95% CI = 1.116–5.856, *p* = 0.026), WBC (OR = 1.195, 95% CI = 1.084–1.317, *p* < 0.001), neutrophils (OR = 1.233, 95% CI = 1.111–1.368, *p* < 0.001), eosinophils (OR = 0.003, 95% CI = 0–0.168, *p* = 0.040), and calcium (OR = 0.098, 95% CI = 0.011–0.901, *p* = 0.040), were significantly correlated with disease severity. However, age, hypertension, diabetes, APTT, fibrinogen, TT, blood RBC, hemoglobin, platelets, lymphocytes, monocytes, basophil count, and CRP level were not significantly correlated with disease severity. In the multivariate model, D-dimer level (OR = 2.631, 95% CI = 1.018–6.802, *p* = 0.046) and neutrophils (OR = 1.2, 95% CI = 1.07–1.345, *p* < 0.001) were independent risk factors for disease severity. WBC were excluded from the multivariate analysis due to the strong correlation between WBC and neutrophils (*r* = 0.933, *p* < 0.001; Table 4).

**Table 4:** Univariate and multivariable logistic regression models of disease severity.

	**Univariate analysis**		**Multivariable analysis**	
	**OR (95% CI)**	***p***	**OR (95% CI)**	***p***
Age at onset	0.993 (0.973–1.013)	0.493		
Gender	1.906 (1.015–3.579)	0.045^*^	1.644 (0.80–3.378)	0.176
Hypertension	0.491 (0.157–1.537)	0.222		
Diabetes	0.938 (0.129–6.828)	0.950		
PT	1.332 (1.027–1.726)	0.031^*^	1.109 (0.823–1.494)	0.496
APTT	0.994 (0.954–1.036)	0.777		
Fibrinogen	1.004 (0.814–1.238)	0.971		
TT	0.967 (0.826–1.131)	0.672		
D-dimer	2.557 (1.116–5.856)	0.026^*^	2.631 (1.018–6.802)	0.046^*^
WBC	1.195 (1.084–1.317)	<0.001^*^		
RBC	0.733 (0.405–1.328)	0.306		
Hemoglobin	0.987 (0.968–1.006)	0.170		
Platelet	0.999 (0.995–1.003)	0.627		
Neutrophil	1.233 (1.111–1.368)	<0.001^*^	1.2 (1.07–1.345)	0.002^*^
Lymphocyte	0.865 (0.680–1.100)	0.236		
Monocytes	0.892 (0.623–1.276)	0.531		
Eosinophils	0.003 (0–0.168)	0.005^*^	0.099 (0.002–5.024)	0.249
Basophil	0.004 (0–14.446)	0.189		
Calcium	0.098 (0.011–0.901)	0.040^*^	0.328 (0.038–2.799)	0.308
CRP	1.018 (0.988–1.049)	0.249		

WBC were not included in the multivariate analysis because of the strong correlation between WBC and neutrophils (r = 0.933, *p* < 0.001).

CI, confidence interval; PT, prothrombin time; APTT, activated partial thrombin time; TT, thrombin time; WBC, white blood cell; RBC, red blood cell; CRP, C-reaction protein.

* *p* < 0.05.

**Figure 2:**
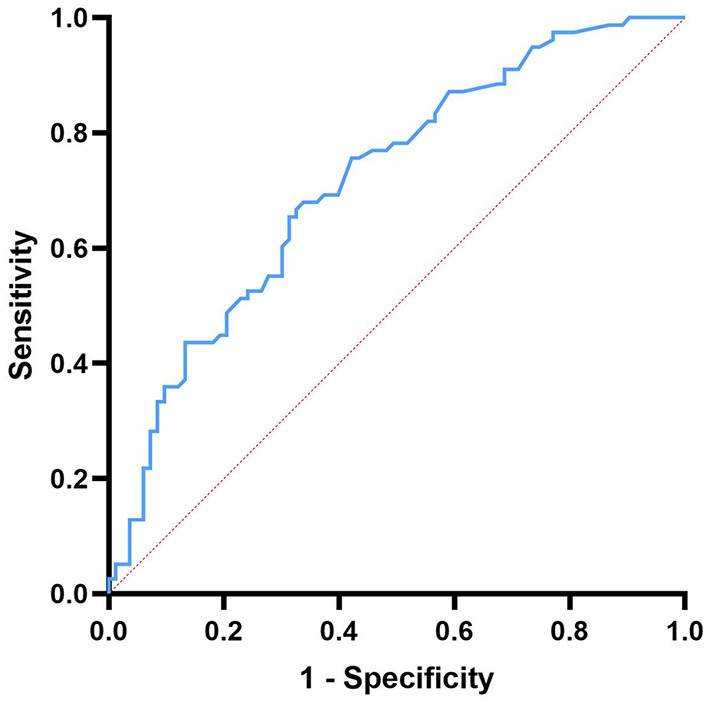
Receiver operating characteristic curve showing the predictive ability of D-dimer for disease severity.

The ROC curve was used to analyze the predictive value of D-dimer levels for disease severity. The area under the curve was 0.716 (95% CI = 0.64–0.80, *p* < 0.001), the best cut-off value was D-dimer = 0.147, the sensitivity was 0.651, and the specificity was 0.705, showing a good predictive ability for disease severity (Figure 2).

### Correlation between D-dimer levels and other laboratory indicators in patients with anti-NMDAR encephalitis

Spearman correlation analysis showed that age (*r* = 0.297, *p* < 0.001), APTT levels (*r* = −0.318, *p* < 0.001), fibrinogen (*r* = 0.253, *p* = 0.001), WBC (*r* = 0.294, *p* < 0.001), RBC (*r* = −0.175, *p* = 0.027), hemoglobin (*r* = −0.183, *p* = 0.020), neutrophils (*r* = 0.301, *p* < 0.001), lymphocytes (*r* = −0.292, *p* < 0.001), basophils (*r* = −0.170, *p* = 0.031), calcium (*r* = −0.249, *p* = 0.002), and CRP (*r* = 0.265, *p* = 0.002) were significantly correlated with D-dimer levels in anti-NMDAR encephalitis. There was no obvious correlation between gender and PT, platelets, monocytes, eosinophils, and D-dimer levels (*p* > 0.05; Table 5).

**Table 5:** Spearman correlation between D-dimer levels and other Laboratory indicators in patients with anti-NMDAR encephalitis.

	***r***	***p***
Age at onset	0.297	<0.001^*^
Gender	0.087	0.272
PT	0.011	0.89
APTT	−0.318	<0.001^*^
Fibrinogen	0.253	0.001^*^
TT	−0.008	0.921
WBC	0.294	<0.001^*^
RBC	−0.175	0.027^*^
Hemoglobin	−0.183	0.020^*^
Platelet	−0.110	0.163
Neutrophil	0.301	<0.001^*^
Lymphocyte	−0.292	<0.001^*^
Monocytes	0.065	0.416
Eosinophils	−0.109	0.165
Basophil	−0.170	0.031^*^
Calcium	−0.249	0.002^*^
CRP	0.265	0.002^*^

Anti-NMDAR encephalitis, anti-N-methyl-D-aspartate receptor encephalitis; PT, prothrombin time; APTT, activated partial thrombin time; TT, thrombin time; WBC, white blood cell; RBC, red blood cell; CRP, C-reaction protein.

* *p* < 0.05.

## Discussion

Previous studies have demonstrated the role of D-dimer levels in the severity of immune-related diseases. In this study, we compared the differences in routine blood test results and coagulation function between healthy individuals and patients with anti-NMDAR encephalitis. We evaluated the effect of D-dimer on disease severity in anti-NMDAR encephalitis. We discovered that high D-dimer and neutrophil levels were associated with a more severe disease at onset. D-dimer levels were positively correlated with neutrophils, leukocytes, and CRP and negatively correlated with eosinophil and calcium levels in patients with anti-NMDAR encephalitis. Furthermore, D-dimer levels were found to be an independent predictor of the severity of anti-NMDAR encephalitis.

This study showed that patients with anti-NMDAR encephalitis had shorter APTT and higher D-dimer levels, and the latter was correlated with disease severity. Anti-NMDAR encephalitis is an autoimmune mediated inflammatory disease. Inflammatory phenomena that occur in anti-NMDAR encephalitis are closely associated with a prothrombotic state. Previous studies have shown that systemic inflammation is a potent prethrombotic stimulus and inflammatory mechanisms contribute to activating coagulation factors, reducing natural anticoagulants, and inhibiting fibrinolytic activity (13). Fibrinogen levels increase in inflammation conditions because they promote fibrinogen synthesis, unless consumptive coagulopathy occurs (14). Patients with anti-NMDAR encephalitis had elevated fibrinogen levels in this study, which is consistent with the above results. This may indicate that higher D-dimer levels in patients with anti-NMDAR encephalitis are associated with inflammation rather than thrombosis.

In this study, neutrophil levels were significantly increased in patients with NMDAR encephalitis, which correlated with disease severity and D-dimer levels. Neutrophils, as an important part of innate and adaptive immunity, affect the maturation and function of other leukocytes by secreting cytokines or cell–cell contact, and play an important role in disease occurrence and progression, including infections, chronic inflammation, tumor and autoimmunity (15, 16). Massberg et al. showed that neutrophils prevent tissue dissemination of pathogens by increasing fibrin formation (17). Studies reported that neutrophil extracellular vesicles and neutrophil extracellular traps (NETs) contribute to thrombosis (18). Kamba et al. demonstrated that neutrophils release tissue factors through NETs, induce thrombin generation, and promote hypercoagulation in vasculitis (19, 20). Recent observations have demonstrated that NETs were blocked by therapeutic-dose low-molecular-weight heparin in coronavirus disease 2019 (21). These findings may provide new therapeutic strategies for the treatment of anti-NMDAR encephalitis.

Inflammatory cytokines induce apoptosis and eosinophil degranulation, causing excessive loss of eosinophils (22). Eosinophils secrete diverse proteins in response to stimuli, including eosinophil cationic protein, which produces endogenous thrombin and promotes thrombus formation (23). In patients with NMDARs, eosinophil levels were lower, which may be associated with hypercoagulability. Eosinophils are the main storage site of tissue factor in blood vessels, and tissue factor is the initiation factor of the extrinsic coagulation pathway (24). Patients in the severe group had lower eosinophil levels and prolonged PT. Eosinophils were consistently negatively correlated with PT in patients with anti-NMDAR encephalitis. Eosinophils can induce the activation of M2 microglia by secreting IL-4 and IL-13 to promote the elimination of inflammation and play the neuroprotective role of microglia (25). This may explain why patients in the severe group had lower eosinophil levels.

Previous studies have shown that the cytokines produced by systemic inflammatory responses can lead to hypocalcemia (26, 27). The hypocalcemia effect may be caused by upregulation of the calcium-sensing receptor by IL-1β and IL-6 (28–30). In this study, patients with severe forms of the disease had higher inflammation levels and lower blood calcium levels than those in the mild-to-moderate group. Calcium is a key component of the coagulation cascade (31). According to our results, serum calcium level was negatively correlated with PT and D-dimer level in patients with anti-NMDAR encephalitis. However, serum calcium levels are closely related to vitamin D and serum albumin levels and are also affected by the concentration of other ions. Thus, its significance in anti-NMDAR encephalitis requires further study.

Our study has some limitations. First, this was a single-center retrospective study. Therefore, our results should be confirmed in a large, multicenter study. In addition, owing to the limitations of retrospective studies, other laboratory indicators such as fibrinogen degradation products and coagulation factor could not be studied in relation to disease severity and inflammatory response. Finally, the exact mechanism underlying the elevation of D-dimer levels in patients with anti-NMDAR encephalitis, particularly whether it is a mere result of inflammation or a part of disease pathogenesis remains unclear. Anticoagulant therapy has been explored in patients with central nervous system autoimmune diseases (32).

## Conclusion

D-dimer and neutrophil levels can be used as predictors of anti-NMDAR encephalitis severity. These factors can help clinicians identify severely ill patients early enough to take appropriate treatment measures. This study indicated that D-dimer may be considered an effective biomarker for anti-NMDAR encephalitis. The mechanism underlying the effect of serum D-dimer levels on disease progression requires further elucidation.

## Data availability statement

The original contributions presented in the study are included in the article/Supplementary material, further inquiries can be directed to the corresponding authors.

## Ethics statement

The studies involving human participants were reviewed and approved by the Ethics Committee of Zhengzhou University. Written informed consent to participate in this study was provided by the participants' legal guardian/next of kin.

## Author contributions

YSh contributed to conception and design of the research and wrote the first draft of the manuscript. JD, YSo, and YL organized the database. LJ, ZG, RD, and YSh performed the statistical analysis. YY, YJ, and SJ undertook the task of revising the manuscript critically. All authors contributed to manuscript revision, read, and approved the submitted version.

## Conflict of interest

The authors declare that the research was conducted in the absence of any commercial or financial relationships that could be construed as a potential conflict of interest.

## Publisher's note

All claims expressed in this article are solely those of the authors and do not necessarily represent those of their affiliated organizations, or those of the publisher, the editors and the reviewers. Any product that may be evaluated in this article, or claim that may be made by its manufacturer, is not guaranteed or endorsed by the publisher.

## Supplementary material

Supplementary Table 1 Anti-NMDAR encephalitis_Data.

Supplementary Table 2 Healthy individuals_Date.
